# Preferentially Expressed Antigen of Melanoma Prevents Lung Cancer Metastasis

**DOI:** 10.1371/journal.pone.0149640

**Published:** 2016-07-08

**Authors:** Quan Huang, Haifeng Wei, Zhipeng Wu, Lin Li, Liangfang Yao, Zhengwang Sun, Lei Li, Zaijun Lin, Wei Xu, Shuai Han, Wenjiao Cao, Yunfei Xu, Dianwen Song, Xinghai Yang, Jianru Xiao

**Affiliations:** 1 Shanghai Key Laboratory of Regulatory Biology, Institute of Biomedical Sciences, School of Life Sciences, East China Normal University, 500 Dongchuan Road, Shanghai 200241, China; 2 Department of Orthopedic Oncology, Changzheng Hospital, The Second Military Medical University, No. 415 Fengyang Road, Shanghai, 200003, China; 3 The International Peace Maternity& Child Health Hospital of China welfare institute (IPMCH), Shanghai, China; 4 Urology Department, Tenth People's Hospital of Tongji University, Shanghai, PR China; University of South Alabama Mitchell Cancer Institute, UNITED STATES

## Abstract

Lung cancer is the most common cause of cancer death worldwide. The poor survival rate is largely due to the extensive local invasion and metastasis. However, the mechanisms underlying the invasion and metastasis of lung cancer cells remain largely elusive. In this study, we examined the role of preferentially expressed antigen of melanoma (PRAME) in lung cancer metastasis. Our results show that PRAME is downregulated in lung adenocarcinoma and lung bone metastasis compared with normal human lung. Knockdown of PRAME decreases the expression of E-Cadherin and promotes the proliferation, invasion, and metastasis of lung cancer cells by regulating multiple critical genes, most of which are related to cell migration, including MMP1, CCL2, CTGF, and PLAU. Clinical data analysis reveals that the expression of MMP1 correlates with the clinical features and outcome of lung adenocarcinoma. Taken together, our data demonstrate that PRAME plays a role in preventing the invasion and metastasis of lung adenocarcinoma and novel diagnostic or therapeutic strategies can be developed by targeting PRAME.

## Introduction

Lung cancer is the leading cause of cancer-related mortality in the world. About 220,000 new cases and 160,000 deaths from lung cancer were reported in the United States in 2014 [1]. Lung adenocarcinoma, belonging to non-small cell lung cancer (NSCLC), is the most common type of lung cancer accounting for more than 50% of NSCLC. Like other cancer types, lung adenocarcinoma has an extremely poor prognosis once it has progressed to the metastatic stage. The 5-year relative survival rate for those diagnosed with lung cancer that has metastatic tumors is about 2%, far less than those without metastasis. Development of novel strategies for the prevention of metastasis helps people to live longer and increase their quality of life.

Metastasis is the spread of tumor cells to tissues and organs other than where it is originated and the formation of new tumors. The metastatic cascade is composed of three main processes: invasion, intravasation, and extravasation. A large number of molecular and cell-biological events are involved in each of these processes [2]. Epithelial-mesenchymal transition (EMT) is an early and key step in metastatic cascade, which is regulated by multiple signaling pathways, including but not limited to transforming growth factor-β (TGF-β) and epidermal growth factor (EGF) [3]. The hallmark of EMT is the decrease of E-cadherin expression, which is a calcium-dependent cell-cell adhesion glycoprotein. Loss of E-cadherin decreases the strength of cellular adhesion and cellular polarity of epithelial cells and promotes the migration and invasion, assuming the phenotypes of mesenchymal cells [4]. The expression of E-cadherin is under the control of a variety of signaling molecules. For example, snail family zinc finger 1 (SNAI1), snail family zinc finger 2 (SNAI2), zinc finger E-box–binding homeobox 1 (ZEB1), zinc finger E-box–binding homeobox 2 (ZEB2), delta-crystallin/E2-box factor 1 (DeltaEF1), and twist basic helix-loop-helix transcription factor 1 (TWIST1) have been shown to downregulate E-cadherin expression [5, 6]. On the other hand, some other genes, such as AML1, Sp1, p300, and HNF3 upregulate the expression of E-cadherin in breast cancer [7]. Though great efforts have been made to gain insights into the mechanisms underlying lung cancer metastasis cascades, effective strategies for the prevention of lung cancer metastasis are still lack.

The preferentially expressed antigen of melanoma (PRAME) was initially identified as a tumor-associated antigen recognized by cytotoxic T lymphocytes against a melanoma surface antigen [8]. PRAME has been widely studied and emerged as a marker of disease activity and prognosis in leukemia and breast cancer [9–11]. In consistent with these studies, a previous study has shown that PRAME is also expressed in lung cancers [12]. However, the functional roles of PRAME in lung cancer development remain largely unrevealed.

In this study, we demonstrated that the expression of PRAME and E-cadherin was decreased in the human lung adenocarcinoma and lung bone metastases. Moreover, knockdown of PRAME decreased the expression of E-cadherin and promoted the proliferation of lung cancer cells PC9 and A549. The migration and invasion of lung cancer cells were enhanced after the PRAME knockdown. Furthermore, RNA-sequence analysis revealed that cell migration-related genes, including MMP1, CTGF, CCL2, and PLAU, were upregulated in PC9 cells transfected with PRAME siRNA. Finally, clinical data analysis showed that the expression of MMP1 correlated with the stage, recovery, and modality of lung cancer patients. Taken together, our data suggest that PRAME serves as a tumor suppressor of lung adenocarcinoma via downregulating E-cadherin and MMP1-mediated migration, leading to the inhibition of EMT. Novel strategies might be developed to prevent EMT and metastasis of lung adenocarcinoma by targeting PRAME.

## Materials and Methods

### Ethics Statement

This study was carried out in strict accordance with the recommendations in the Guide for the Care and Use of Laboratory Animals of Shanghai Changzheng Hospital. The protocol was approved by the Committee on the Ethics of Animal Experiments of the Shanghai Changzheng Hospital (Permit Number: 2014SL028). All surgery was performed under sodium pentobarbital anesthesia, and all efforts were made to minimize suffering.

### Cell culture and siRNA transfection

PC9 and A549 cells originally purchased from American Type Culture Collection (ATCC) were kindly provided by Dr. Cai in the Second Military Medical University in China. Cells were cultured in RPMI and MEM, both of which were supplemented with 10% fetal bovine serum (FBS), 2 mmol/l glutamine, 100 units/mL penicillin and 100 μg/mL streptomycin. Cells were cultured in a humidified atmosphere of 95% air and 5% CO2 at 37°C. For transfection of siRNA, Lipofectamine® RNAiMAX Reagent (Invitrogen) was mixed with the siRNA construct according to the manufacturer’s instructions and added to PC9 or A549 cells in 24-well plate. Pre-designed SMARTpool ON-TARGETplusAccell PRAME siRNA duplexes were designed and synthesized by Dharmacon (Lafayette, CO).

### MTT assay

PC9 and A549 cells were seeded in 96-well microplates with 2500 cells/well and incubated for 24 h in 100 μl culture medium. The cells were then treated with control or PRAME siRNA for 48 hr. MTT [100 μl (5 g/l)] was added to the cells which were then cultivated for another 4 hr. Following the removal of the supernatant, DMSO (100 μl/well) was added to the cells which were agitated for 15 min. The absorbance was measured at 570 nm by an ELISA reader. Each assay was repeated three times.

### Western blot

Whole-cell lysates from PC9 and A549 cells were prepared and 40 μg of protein was separated using 10% SDS-PAGE gel. After blocking with TBST containing 5% skim milk or 3% bovine serum albumin (Sigma, St. Louis, MO), blots were incubated at 4°C overnight with the primary antibodies against human PRAME protein (1: 500), E-Cadherin (1: 2000) and actin (1: 5000), respectively. A horseradish peroxidase conjugated IgG was used as secondary antibody according to manufacturer’s instruction. The density of the band of interest was measured using densitometric measurement with Imge J (NIH) and normalized to actin.

### Quantitative reverse transcriptase-polymerase chain reaction (qRT-PCR)

The total RNA from PC9 or A549 cells was prepared using the Trizol reagent (Invitrogen, Carlsbad, CA). 1 μg of total RNA was treated with DNase I (Invitrogen) and the cDNA was synthesized *in vitro* from the mRNA template using SuperScript® III First-Strand Synthesis Kit (Invitrogen). cDNA was amplified by PCR using specific primer pairs for PRAME. The sequences of primers are as follows: PRAME sense (5’-3’: CAGGACTTCTGGACTGTATGGT) and PRAME antisense (5’-3’: CTACGAGCACCTCTACTGGAA). Actin served as the internal control. The PCR products were subjected to agarose gel electrophoresis and detected using gel imaging analysis machine (Tanon 2500).

### Invasion assay

Lung cancer cell invasion was measured by using the Matrigel-coated transwell culture chambers as described previously [13]. Briefly, PC9 or A549 cells prepared in serum-free 1640 medium were transfected with control or PRAME siRNAs and plated in the upper chamber of transwell chambers. The lower chamber was filled with 10% FBS-containing medium. Cells were incubated for 24h at 37°C in a humidified atmosphere with 95% air and 5% O_2_. The invasive cells penetrated through the Matrigel in the lower chamber were fixed and photographed using a light microscope for quantification.

### Mouse model of bone metastasis

All experiments were performed according to guidelines of animal ethical committee of Changzheng Hospital. Subconfluent tumor cells were harvested, washed and prepared in PBS. The female BALB/c nude mice at the age of 5 weeks were anesthetized and placed in the supine position. With a 29-gauge needle, PC9 cells expressing the luciferase reporter gene were implanted into nude mice at 2×10^6^cells in 100 μl per spot. Twenty days after injection, mice bearing tumors were euthanized by CO2 asphyxiation and tissues were collected for immunohistological examination. Mice were euthanized when tumors exceeds 100 mm^3^. For Bioluminescence images assay, mice were intraperitoneally injected with D-luciferin(200 mg/kg) and anesthetized with isoflurane 12 min postD-luciferin injection. The BLI images were collected with an IVIS imaging system (Xenogen, Alameda, CA). X-ray photographs were taken (Faxitron,USA). Both intratibial and intracardiac injection of cancer cells have been utilized for generating bone metastasis animal model, though neither of them recapitulate early steps of bone metastasis process [14, 15]. However, we tried to use the intracardiac injection which contains the extravasation step, but did not succeed.

### RNA sequencing and differentially expressed genes (DEGs) analysis

Three control and three FOXD3 knockdown A549 cell lines were subjected to RNA sequencing. With the RNA sequencing data prepared, we first mapped the sequencing tags to the human (Homo sapiens) genome (version hg19) using TopHat [16], then the expression abundance (FPKM) value of each gene was estimated by running cufflinks [17] and the differentially expressed genes were assessed by Cuffdiff. Only those genes with |fold change|> 2 and adjusted p value < 0.01 were recognized as statistically differentially expressed between two groups. The adjusted p value was obtained through applying Benjamini and Hochberg's (BH) false discovery rate correction on the original p value, and fold change threshold was selected based on our purpose of focusing on significantly differentially expressed genes.

### Hierarchical clustering

We performed hierarchical clustering [18] to classify analyzed samples based on gene expression profiles. Hierarchical clustering was carried out using differentially expressed genes to observe the global gene expression patterns. Besides, the DEGs, which were classified in specific biological processes (Gene Ontology terms) and KEGG pathways, were further extracted and the expression pattern of those DEGs was characterized, and heatmaps for the DEGs classified in targeted biological processes or KEGG pathways using R package.

### GO and KEGG Pathway analysis

We utilized R packages–GO.db, KEGG.db and KEGGREST to detect Gene Ontology categories and KEGG pathways with significant enrichment in DEGs comparing to which across all measured genes. The significantly enriched biological processes were identified as p value less than threshold value 0.01. As to KEGG pathway, p value was set to less than 0.05.

### Construction of biological network

We downloaded protein-protein interaction (PPI) databases from HPRD [19], BIOGRID [20], and PIP [21] databases. Pair interactions, which were included in any of the three databases, were chosen to be included in our curated PPI database. As a result, 561405 pair interactions were in our database. Cytoscape [22] was utilized to construct interaction network. Interacted gene pairs existed in our curated PPI database was imported as stored network. After functional enrichment analysis, the DEGs specified in dramatically altered biological processes (Gene Ontology terms) and KEGG pathways were mapped to corresponding networks respectively to analyze interaction.

### Clinical outcome analysis

The clinical data of Lung Adenocarcinoma (LUAD) patients were obtained through TCGA pan-cancer synapse (https://www.synapse.org/#!Synapse:syn300013). To investigate the clinical relevance of a specific gene, samples were divided into two groups based on the relative RNA expression of target gene, and then the vital status and survival length recorded in clinics were incorporated to perform the Kaplan-Meier survival analysis (use “Survival” package in R), log-rank test was utilized in determining the difference of survival between two groups of patients. Normal human lung tissue, human lung adenocarcinomaand lung bone metastasis were from Changzheng Hospital.

### Statistical analysis

Data are presented as mean ± s.e.m. of three independent experiments. Significance of means between two groups is determined by student’s t-test. Difference in cell growth between control and PRAME siRNA-treated groups was evaluated by repeated measures analysis of variance (ANOVA). A P-value of < 0.05 was considered significantly different.

## Results

### Inhibition of PRAME promotes the proliferation and invasion of lung cancer cells

**Fig 1:**
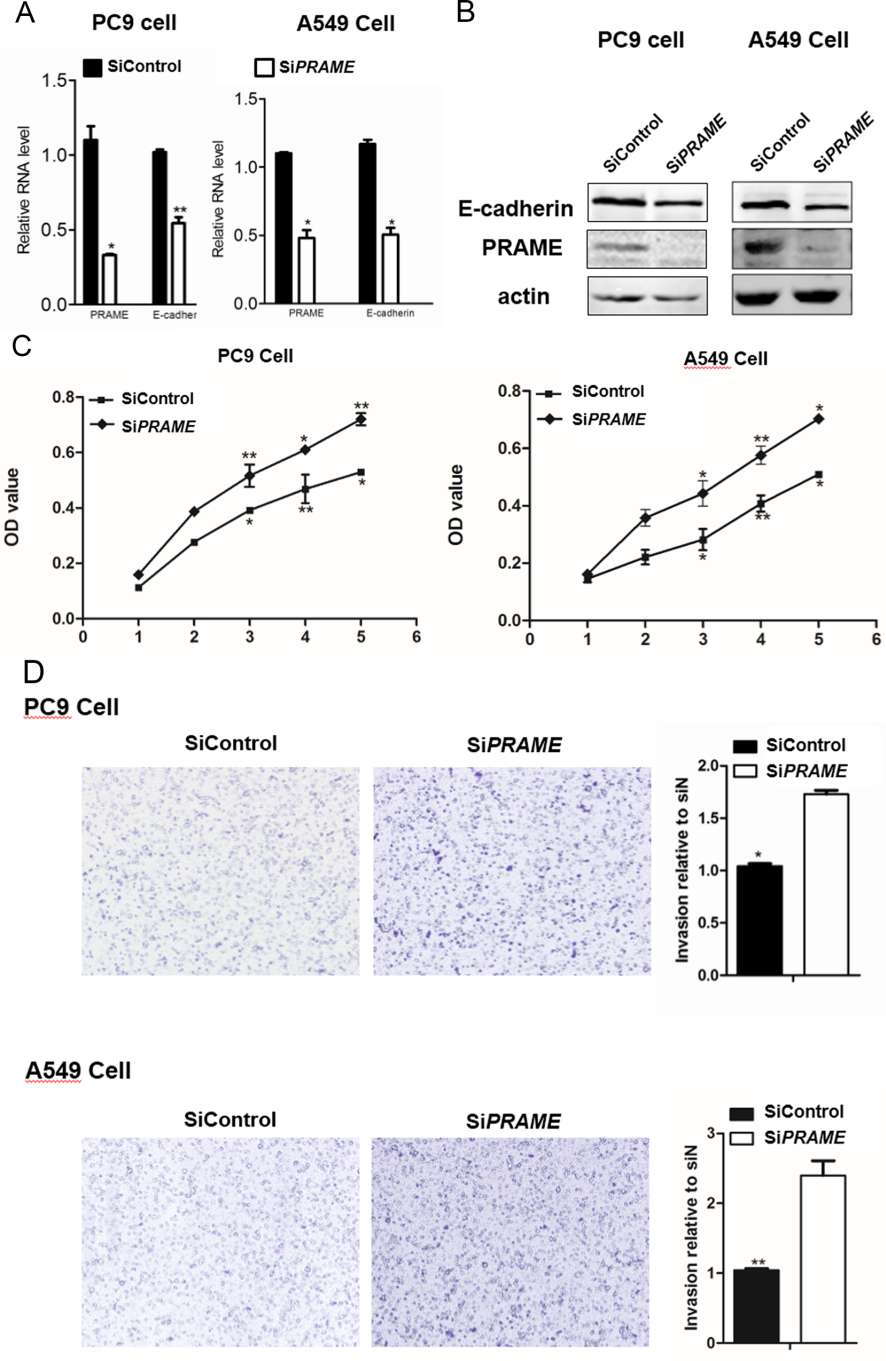
Cell proliferation and invasion of PRAME siRNA-treated cells. RT-PCR(A) and western blot (B) show that PRAME siRNA transfected PC9 and A549 cells exhibited decreased PRAME and E-cadherin expression. Actin serves the loading control in western blot experiments. (C) MTT assay of PC9 and A549 cells after PRAME and control siRNA transfection. (D) Pictures and bar graphs show the increased invasion of lung cancer cells after PRAME knockdown. * p<0.05, ** p<0.001.

Cancer cell proliferation and invasion are important steps in metastasis. To investigate the role of PRAME in lung cancer cell progression, we examined the effect of PRAME knockdown on lung cancer cell proliferation and invasion. As shown in Fig 1A & 1B, 3 days after the PRAME siRNA transfection, the PRAME expression was significantly reduced in both PC9 and A549 cells compared with that in control siRNA-transfected cells as evidenced by RT-PCR and western blot, indicating the successful knockdown of PRAME in these cells. After the knockdown of PRAME, the proliferation of both PC9 and A549 cells was significantly increased starting at 3 days after transfection as evidenced by MTT assay, suggesting that PRAME inhibits lung cancer cell proliferation (Fig 1C). Next, we continued to examine the effect of PRAME on invasion of lung cancer cells. As shown in Fig 1D, the number of cells penetrated through the Matrigel was dramatically increased in both PC9 and A549 cells when transfected with PRAME siRNA, indicating that PRAME inhibits the invasion of lung cancer cells. In line with its roles in inhibiting cancer cell growth and invasion, knockdown of PRAME dramatically reduced the expression of E-cadherin (Fig 1A & 1B), an important gene known to suppress tumor progression.

### Differential gene expression after knockdown of PRAME

**Fig 2:**
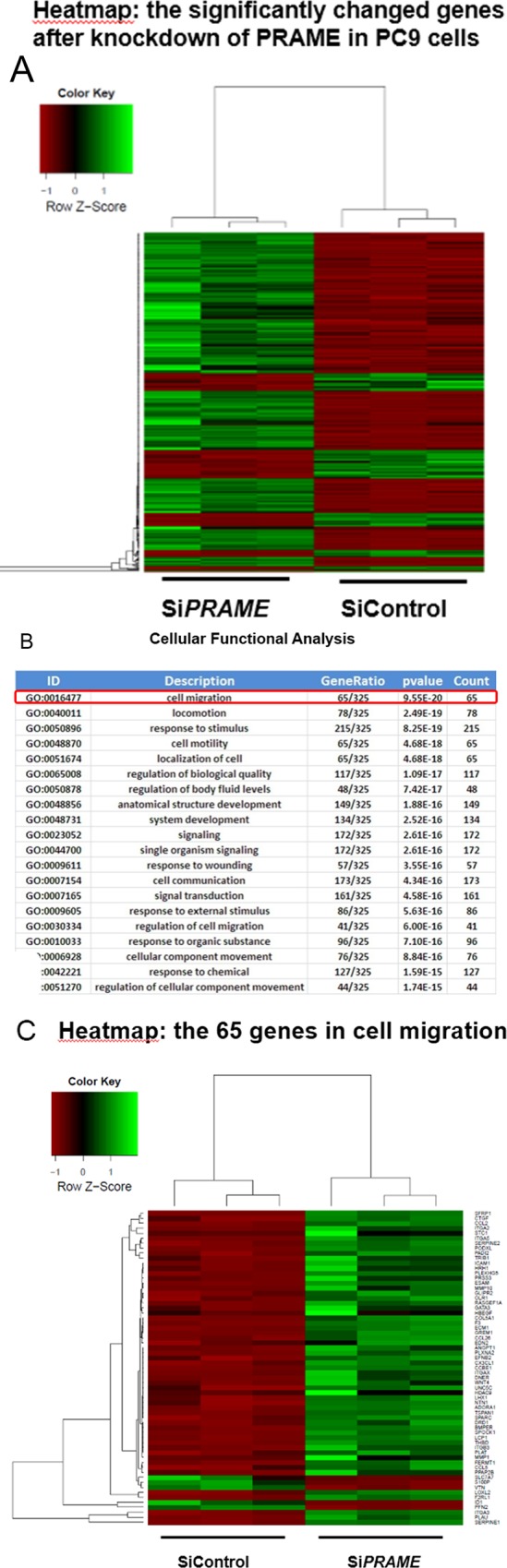
RNA-seq profiling of PC9 cells after PRAME knockdown. (A) RNA-seq analysis of 3 samples of PC9 cell transfected with PRAME siRNA and control siRNA. Heatmap represents the differentially expressed genes after the knockdown of PRAME. Each column represents one sample, and each row refers to one gene. Top-left is the color legend with the green color indicating the upregulated genes. (B) GO analysis of the differentially expressed genes for the cellular function characterization of these genes. (C) List of the top 65 genes significantly altered after the knockdown of PRAME, which are closely related to cell migration.

To further examine the role of PRAME on lung cancer cell development, we performed RNA-seq analysis in PC9 cells transfected with control or PRAME siRNA. In this study, 368 genes were detected to be differentially expressed in PC9 cells after PRAME knockdown compared with the control. As shown in the heatmap, the gene expression profiles of PC9 cell transfected with PRAME siRNA clustered together and were distinguishable from that of control siRNA-treated cells (Fig 2A). To elucidate the roles of these genes, we further performed GO enrichment analysis. The result demonstrated that the DEGs were categorized into 20 functional categories. Most of the DEGs were involved in the biological function of cell migration as it ranked on the top function altered by the knockdown of PRAME (Fig 2B). Fig 2C listed the total of 65 genes closely related to cell migration that were significantly changed. These results indicate that PRAME play important roles in the regulation of cancer cell migration.

### Suppression of PRAME increases bone metastases

**Fig 3:**
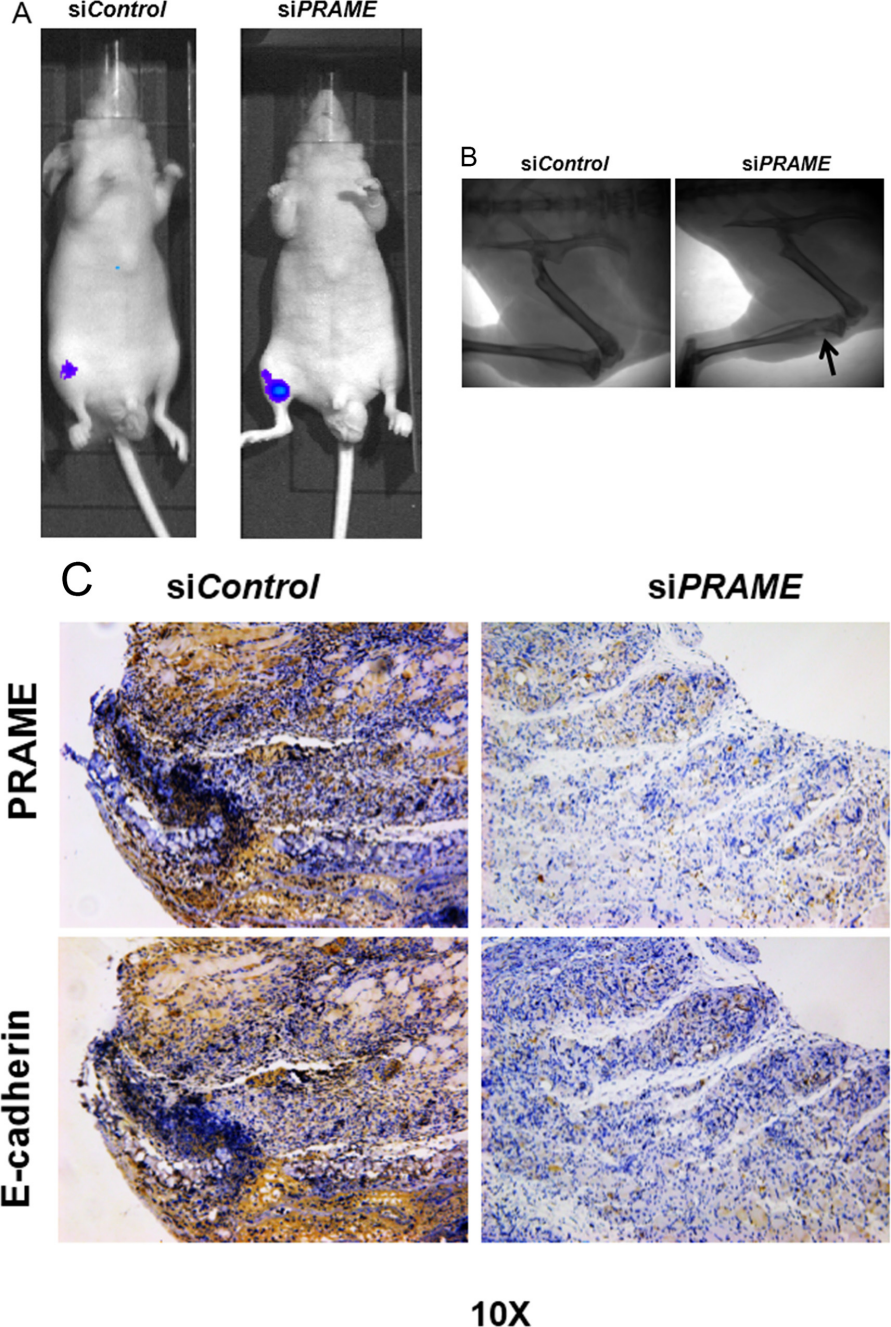
PRAME shows similar expression pattern with E-cadherin in the bone metastasis mouse model. (A) Representative images showing the luminescence signal in xenografted mice after PC9 cells inoculation. (B) X-ray photographs show the osteolytic lesion produced by PRAME siRNA transfected PC9 cells. (c) Histological staining of tibias shows the control siRNA and PRAME siRNA treated tumor cells metastasized to tibial bone.

To examine the role of PRAME in metastatic potential of PC9 cells *in vivo*, we utilized the mouse model of bone metastasis by injecting PC9 cells into the tibia of nude mice. As shown in Fig 3A, we were able to detect luminescence in tibias 20 days after tumor cell inoculation, reflecting the presence of tumor in the bone. More importantly, the luminescence was increased when PRAME siRNA transfected PC9 cells were injected, indicating the increased metastasis of PC9 cells with PRAME knocked down (Fig 3A). The increased metastatic potential was further confirmed by the X-ray photographs showing osteolytic lesion (Fig 3B). As mentioned above, E-Cadherin plays important roles in cell-cell adhesion and reduced expression of E-Cadherin serves as the hallmark of invasive carcinomas [4]. In line with this, our histological examinations revealed that PC9 tumor cells were present in the tibia, where PRAME shared the similar distribution pattern with that of E-Cadherin. After the knockdown of PRAME, the number of PC9 cells presented in the tibia was dramatically increased (Fig 3C). These results demonstrate that PRAME plays a protective role in the prevention of bone metastasis of lung cancer cells.

### PRAME expression is downregulated in human lung carcinoma and metastases

**Fig 4:**
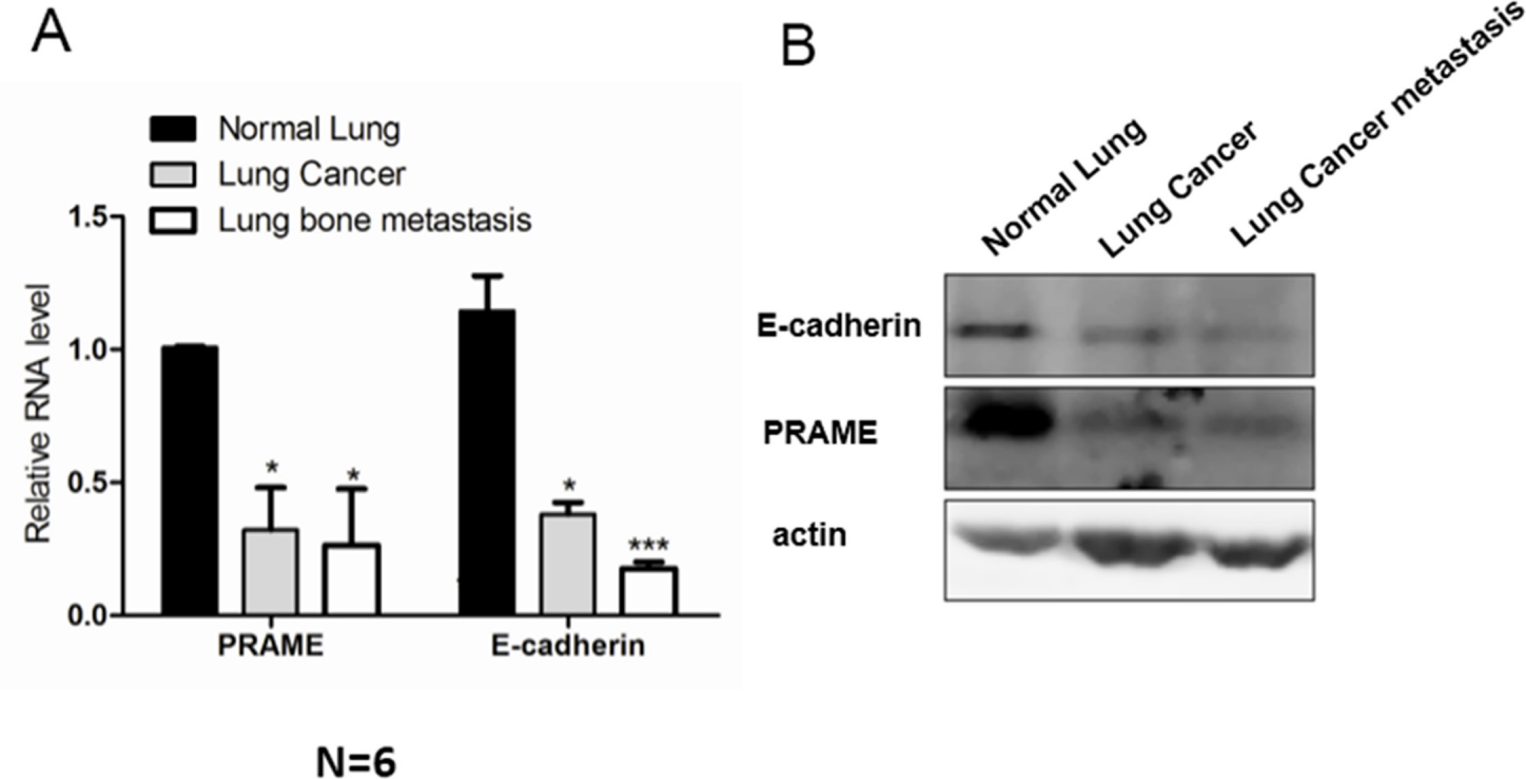
PRAME is down-regulated in the human lung cancer and metastases. RT-PCR (A) and western blot (B) shows that PRAME and E-cadherin are downregulated in lung cancer and lung bone metastasis. Actin serve as the loading control. * p<0.05, *** p<0.0001.

To translate the above *in vitro* results into clinical studies, we examined the expression of both PRAME and E-cadherin in normal human lung tissue, human lung adenocarcinoma, and lung bone metastasis. We performed both RT-PCR and western blot assays in these human tissues. As shown in Fig 4, the mRNA and protein expressions of both PRAME and E-cadherin were down regulated in lung cancer and lung bone metastasis compared with that in normal lung tissue. This study suggests that PRAME can be used as a biomarker or therapeutic target in the diagnosis and prevention of the development of lung metastasis.

### Identification of core genes in cell migration regulated by PRAME

**Fig 5:**
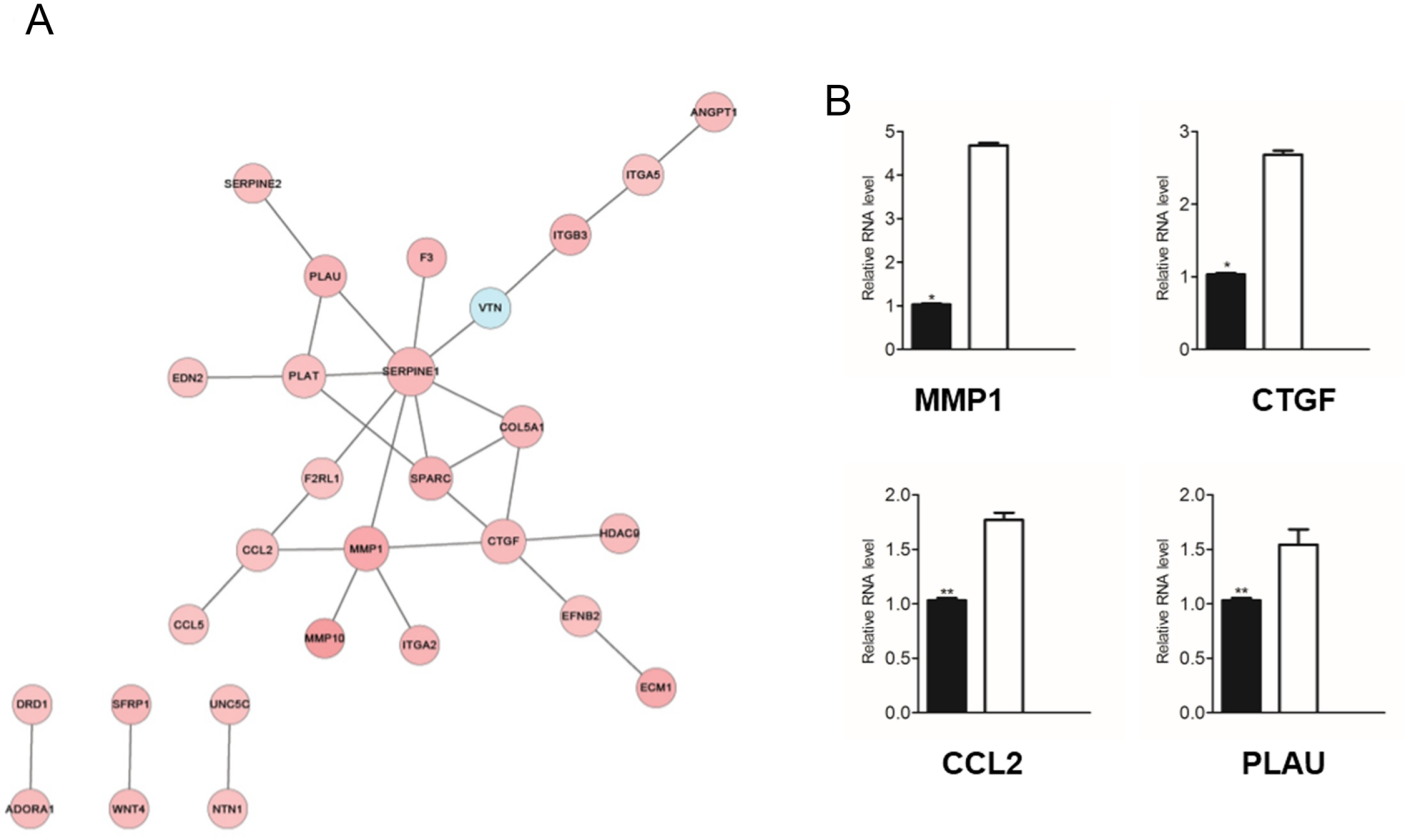
Identification of core genes in cell migration regulated by PRAME. (A) Network analysis predicts the candidate genes in cell migration regulated by PRAME. (B) RT-PCR analysis showing the genes with increased expression in PC9 cells after the transfection of the PRAME siRNA.

To gain insight into the molecular mechanisms underlying the modulation of cell migration by PRAME, we performed protein-protein interaction (PPI) network analysis by downloading databases from HPRD [19], BIOGRID [20], and PIP [21] databases to investigate the relationship among those DEGs regulated by PRAME and how they work coordinately as a molecular group. As shown in Fig 5A, the differentially expressed gene products MMP1, PLAU, CTGF, and CCL2 were connected in the PPI network, indicating that these proteins interacted with each other to form the downstream signaling of PRAME-mediated effects. To further confirm the effect of PRAME on expression levels of MMP1, CTGF, CCL2, and PLAU, we performed RT-PCR analysis. Indeed, our data showed that the expression levels of these genes were significantly upregulated in PC9 cells after knocking down P RAME (Fig 5B).

### MMP1 expression correlates with the lung cancer development

**Fig 6:**
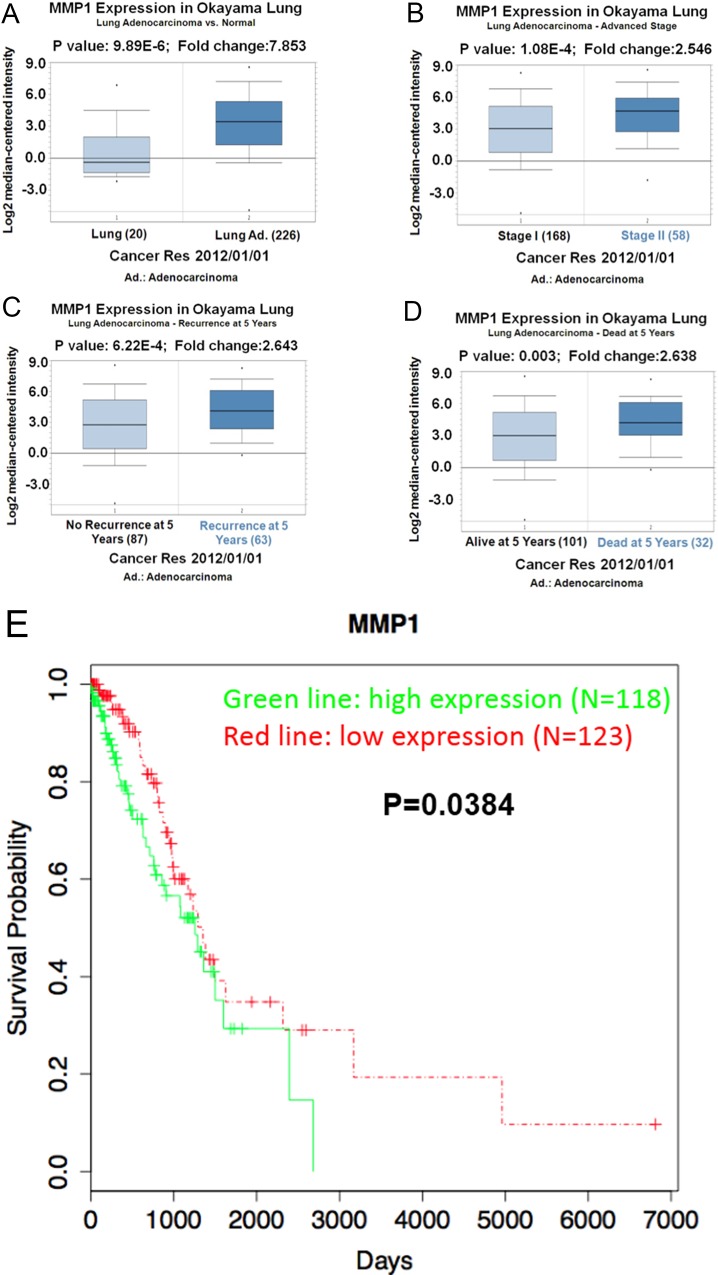
MMP1 correlates with the clinical features of lung cancer. (A) Increased expression of MMP1 is observed in lung adenocarcinoma compared with normal lung tissue. (B) Higher expression of MMP1 in Stage II than in Stage I adenocarcinoma. Increased recurrence (C) and deaths at 5 years (D) correlate with higher expression of MMP1. (E) Survival probability is increased in patients with low MMP1 expression.

After the identification of MMP1 as the downstream gene of PRAME, we further analyzed the clinical relevance of MMP1 in lung adenocarcinoma using the clinical data of lung adenocarcinoma patients. As shown in Fig 6A, the expression of MMP1 was significantly higher in lung adenocarcinoma than in normal lung tissue. It was even higher in Stage II than in Stage I of lung adenocarcinoma (Fig 6B). Both the recurrence and the dead of lung adenocarcinoma patients at 5 years correlated with the expression of MMP1 (Fig 6C & 6D). These results suggest that MMP1 plays important roles in the development of lung adenocarcinoma. Indeed, a higher survival probability was observed in patients with lower MMP1 expression (Fig 6E).

## Discussion

Lung cancer is one of the most common cancers in the world, which accounts for about 27% of all cancer deaths. The high mortality of lung cancer is largely attributed to early metastasis. High proliferation rate of cancer cells is the characteristics of metastasis. In supporting the role of PRAME in lung cancer metastasis, we demonstrated that decrease the expression of PRAME dramatically increased the proliferation of lung cancer cells PC9 and A549. This result indicates that PRAME may play a protective role in lung cancer development. The other major process of metastasis is invasion of cancer cells, which enables the transfer of cancer cells from the origin to other parts of the body. In line with the protection of PRAME in lung cancer, our data showed that knockdown of PRAME increased the invasion of lung cancer cells. Furthermore, cell migration ranks on the top of functional GO terms in GO analysis of DEGs derived from our RNA-seq analysis, indicating the most important function affected by the knockdown of PRAME is the cell migration. Taken together, these data clearly demonstrate that PRAME play important roles in lung cancer development.

It is well known that EMT is the key step in metastasis and the decreased expression of E-cadherin is the hallmark of EMT [4]. Interestingly, we found that the expression of E-cadherin was attenuated in PC9 and A549 cells when PRAME was inhibited, suggesting that PRAME controls E-cadherin expression and EMT development. Using the mouse model of bone metastasis, we further demonstrated that intratibial injection of PC9 cells induced bone metastasis and more importantly the expression of PRAME in bone metastasis shares the same pattern with that of E-cadherin. Knockdown of PRAME in PC9 cells significantly increased the bone metastasis and induced osteolytic lesion. The correlation between PRAME and E-cadherin further supports the functional interaction between them and suggests the similar role of PRAME as E-cadherin in EMT.

As mentioned before, multiple signaling pathways are involved in the regulation of EMT and metastasis cascades. We continued to investigate the molecular mechanisms underlying PRAME-induced prevention of metastasis. We performed PPI network analysis and found that the differentially expressed MMP1, PLAU, CCL2, and CTGF were included in an interaction network. Furthermore, these genes were upregulated when PRAME was inhibited. In consistent with our observations, several lines of evidence indicate that these genes promote metastasis. It is well known that MMP1 promotes the invasion and metastasis of many cancers including lung cancer, breast cancer, and colorectal cancer [23–25]. In line with previous studies and our current *in vitro* studies, the clinical data analysis demonstrates that the expression of MMP1 correlates with the development, recovery, and survival of lung adenocarcinoma patients.

CCL2 has been shown to recruit inflammatory monocytes facilitating the breast cancer metastasis [26], and disinhibition of CCL2 accelerates the breast cancer metastasis [27]. CTGF is a secreted protein expressed in various tissues that can modulate the invasive behavior of certain cancer cells by binding to integrins on the cell surface [28]. The expression of CTGF has been shown to be increased in various cancers, including breast cancer and pancreatic cancer [29, 30]. CTGF-specific antibody attenuates the tumor metastasis of pancreatic cancer [31, 32] and melanoma [33]. The role of PLAU in promoting metastasis has been demonstrated in a previous study showing that knockdown of PLAU decreased the migration and invasion of prostate cancer cells [34]. Combined our PPI network analysis with previous studies, PRAME knockdwon upregulates MMP1, CCL2, CTGF, and PLAU and contribute to lung cancer metastasis.

In summary, our study demonstrates that PRAM is downregulated in lung cancer. Downregulation of PRAME in lung cancer cells promotes the metastasis via E-cadherin signaling pathway. Prometastatic genes MMP1, CCL2, CTGF and PLAU are the downstream events of PRAME-mediated effects. These results suggest that PRAME many play a protective role in lung adenocarcinoma metastasis. The expression level of PRAME could be used as a biomarker in early detection, prognostication and mornitoring for recurrence of lung adenocarcinoma. Drugs promoting the expression of PRAME could be developed to prevent the progression and metastasis of lung cancer.
